# Clinical characteristics and short-term outcomes of adult stroke patients admitted to Jimma Medical Center, Ethiopia: a prospective cohort study

**DOI:** 10.11604/pamj.2023.44.49.37588

**Published:** 2023-01-25

**Authors:** Abel Wubshet, Korinan Fanta, Tadesse Dukesa Gemachu, Addis Birhanu, Esayas Kebede Gudina

**Affiliations:** 1 Department of Internal Medicine, College of Health Science, Wollega University, Nekemte, Oromia, Ethiopia,; 2 Department of Clinical Pharmacy, Institute of Health, Jimma University, Jimma, Oromia, Ethiopia,; 3 Department of Internal Medicine, Institute of Health, Jimma University, Jimma, Oromia, Ethiopia,; 4 Department of Epidemiology, Institute of Health, Jimma University, Jimma, Oromia, Ethiopia

**Keywords:** Cerebrovascular disease, ischemic stroke, hemorrhagic stroke, disability, mortality, sub-Saharan Africa

## Abstract

**Introduction:**

sub-Saharan African countries are facing a rapid increase in stroke incidence and mortality. However, there is a paucity of clinical studies on the burden of stroke and its short-term outcomes. Hence, this study is aimed at evaluating risk factors, clinical characteristics, management, and 28-day clinical outcomes among stroke patients.

**Methods:**

a prospective observational study was conducted at Jimma Medical Center, Ethiopia from July 2020 to January 31^st^, 2021. All adult patients diagnosed with stroke were enrolled consecutively and followed for 28 days starting from admission. Data were analyzed using SPSS version 23 and Multivariable cox regression was used to identify factors associated with 28-day all-cause mortality.

**Results:**

among 153 patients enrolled in this study, 127 (83%) had brain CT-scan and hemorrhagic stroke accounts for 66 (52%). About half 81 (53%) of the participants were male and the mean age was 57 years. Regarding in-hospital management, antihypertensive, statins, and aspirin was given to 80 (52%), 72 (47%), and 68 (44%) patients respectively. The overall in-hospital mortality rate was 26 (17%) and the all-cause 28-day mortality rate was 39 (25.5%). Rural residence [adjusted Hazard Ratio (aHR): 2.93, 95% Confidence Interval (CI): 1.46-5.81], aspiration pneumonia (aHR= 6.57, 95% CI=3.16-13.66) and increased intracranial pressure (aHR= 3.27, 95% CI=1.56-6.86) were associated with 28-day mortality.

**Conclusion:**

the patients admitted to the hospital with stroke diagnosis had high short-term mortality. Strategies focused on increasing timely arrival and evidence-based management of stroke and its complications could improve stroke patient outcomes.

## Introduction

According to the 2016 Global Burden of Diseases Report, cerebrovascular diseases are the second leading cause of mortality worldwide and the major cause of disability-adjusted life years (DALY) [1]. Among these, 85% of stroke-related death and 87% of DALY losses occur in low and middle-income countries (LMIC) [1,2]. Extensive use of preventive therapies, management of risk factors, and evidence-based medications for the management of stroke resulted in a significant decrease in stroke incidence and improve post-stroke survival in high-income countries [3,4]. Nevertheless, LMICs, sub-Saharan African countries in particular are facing a sharp increase in stroke incidence and mortality [5]. A recent systematic review of epidemiological studies showed that the one-month case fatality rate of stroke in sub-Saharan Africa (SSA) was 24% and 33% at one year [6].

Stroke is the third leading cause of cardiovascular disease (CVD) mortality in Ethiopia following ischemic heart disease and rheumatic heart disease [7]. For instance, a hospital-based study in Ethiopia indicated that stroke accounts for 16.7% of all medical admission and 23.6% of all adult medical case death [8]. This burden of stroke will rise in the future as metabolic syndrome, a common risk factor for stroke and CVD is increasing [9,10]. Stroke affects the working-age group in Ethiopia with a mean age of 53 years [11] to 64 years [12] years compared to developed countries where the older population is commonly affected. Furthermore, the proportion of hemorrhagic stroke is high in Ethiopia [8,11,13] contrary to the developed countries where ischemic stroke is the predominant type of stroke.

Although there are several retrospective patient medical card review studies on in-hospital case fatality of stroke in Ethiopia [12,14-20], Only a few studies examined stroke management and outcomes prospectively [8,11,13]. In addition, those prospectively assessed patient outcomes were confined to in-hospital outcomes and they were conducted about five years ago [11,13]. Even though the aforementioned studies have illustrated valuable data on the epidemiology of stroke in Ethiopian hospitals, prospective up-to-date analysis of stroke clinical characteristics, short-term mortality, and physical disability is important since the country is undergoing rapid epidemiological change and an increasing burden of CVD risk factors [7]. Hence, the present study aimed at examining clinical characteristics, in-hospital complication, and 28-day mortality and physical disability among stroke patients admitted to Jimma Medical Center, Southwest Ethiopia.

## Methods

**Study design and setting:** a prospective observational study was conducted at the Stroke Unit of Jimma Medical Center (JMC), Ethiopia from July 1, 2020, to January 31, 2021. JMC is the only tertiary care hospital located in the southwestern part of the country and it serves as a referral centre for about 20 million population catchment areas. The medical centre has over 1,500 health workers and provides clinical services for approximately 15,000 inpatients, 160,000 outpatients, 11,000 emergency cases, and 4,500 deliveries per year. The Stroke Unit of JMC was established with the assistance of a project from the United Kingdom Aid Direct (UKAID) and Tropical Health and Education Trust (THET) in collaboration with the Southampton Hospital in 2015. The hospital is equipped with Computed Tomography (CT) Scanner and MRI.

**Data collection:** the data collection tool was developed by reviewing prior studies on a similar topic and using the WHO Step-Wise approach to stroke surveillance [21]. The data collection tool includes patient demographics, comorbid conditions or risk factors, clinical presentation, management, in-hospital clinical outcomes, and 28-day mortality and physical disability score using a modified Rankin score (mRS). The data collection tool was pre-tested on 5% of the study population before initiating the actual data collection to check for the applicability and consistency of relevant variables. The data was collected by two medical residents after two days of training on the data collection tool and patient interview techniques. The data collectors interviewed patients and extracted relevant information from patients´ medical charts. Study participants were followed from admission to death or four weeks starting from the first day of hospitalization. The patients were followed on daily basis during their hospital stay and followed through telephone calls and appointments after discharge.

**Outcome and validating methods:** mortality within the first 28 days after hospital admission of the patient was considered the primary outcome of the study. Patients were followed from hospital arrival until died in the hospital or four weeks. Death ascertainment was based on physician duty notes if died in the hospital or through a telephone call to family members or caregivers if died after discharge within 28 days of admission. Duration of hospital stay was calculated as the time gap between hospital admission and discharge or in-hospital death. Stroke severity was measured based on the National Institute of Health Stroke Scale (NIHSS) [8,13], and the patient´s level of consciousness was measured using Glasgow Coma Scale (GCS) [8]. Modified Rankin Scale (mRs) was used to measure physical disability [8,13,22]. The definition of terms used is available in supplementary material

**Statistical analysis:** data were analyzed using a statistical package for social science (SPSS) version 23 (IBM, Armonk, NY, USA). Categorical variables were summarized using proportion and chi-square. Continuous variables were described using mean or median depending on the normality distribution of the data and analyzed using Student's t-test or the Mann-Whitney U test accordingly. Multivariable cox-regression was used to identify independent predictors of 28-day all-cause mortality. Variables with a p-value <0.25 on univariate analysis were considered a candidate for multivariable analysis. After checking proportional hazard assumption by plotting residuals against time, multivariable Cox regression was done using the backward stepwise method. Variables with a two-sided p-value < 0.05 were considered statistically significant.

**Ethical considerations:** ethical clearance was obtained from the Institutional Review Board (IRB) of the Institute of Health, Jimma University. Written informed consent was obtained from all the study participants before starting the data collection process. All patients were granted the right to withdraw from the research. The confidentiality and privacy of patients were assured throughout the study period by removing identifiers from data collection tools using different codes. Neither the case records nor the data extracted were used for any other purpose.

## Results

**Sociodemographic characteristics and medical history:** among 153 stroke patients included in this study, 81 (52.9%) of the participants were male and the mean age was 57±14.9 years. The majority of the patients 92 (60.1%) were from urban/semi-urban areas and almost two-thirds of them 93 (60.8%) had no formal education. Hypertension was the most common comorbid condition recorded in about two-thirds of 97 (63.4%) of stroke patients. Dyslipidaemia and diabetes were recorded in 58 (38.2%) and 28 (18.3%) stroke patients respectively. Other risk factors such as smoking were identified only in about 11 (7.2%) of the patients (Table 1).

**Table 1:** demographic and baseline characteristics of adult stroke patients admitted to JMC, Ethiopia

Variables	Ischemic stroke (N=74)	Hemorrhagic stroke (N=79)	All patients (N=153)	P-value
**Age (mean ± SD)**	59.8±15.3	54.7±14.2	57.1±14.9	0.028*
**Sex**				
Male	34 (45.9)	47 (59.5)	81 (52.9)	0.093
Female	40 (54.1)	32 (40.5)	72 (47.1)	
**Residence**				
Rural	26 (35.1)	35 (44.3)	61 (39.9)	0.247
Urban/semi-urban	48 (64.9)	44 (55.7)	92 (60.1)	
**Education status**				
No formal education	53 (71.6)	40 (50.6)	93 (60.8)	0.016*
Elementary school (1 -8)	16 (21.6)	24 (30.4)	40 (26.1)	
Secondary school and above	5 (6.8)	15 (19.0)	20 (13.1)	
**Average monthly income (in ETB)**				
≤2500	30 (40.5)	25 (31.6)	55 (35.9)	0.517
1001-4500	20 (27.0)	25 (31.6)	45 (29.4)	
>4500	24 (32.4)	29 (36.7)	53 (34.6)	
**Risk factors**				
Smoking	4 (5.4)	7 (8.9)	11 (7.2)	0.536
Alcohol	13 (17.6)	16 (20.3)	29 (19.0)	0.672
Hypertension	45 (60.8)	52 (65.8)	97 (63.4)	0.520
Diabetes	15(20.0)	13 (16.5)	28(18.3)	0.542
Dyslipidemia	24 (32.9)	34 (43.0)	58 (38.2)	0.198
Cardiac illness	11 (14.9)	10 (12.7)	20(13.7)	0.692
Prior stroke	2 (2.7)	3 (3.8)	5 (3.3)	-
HIV/AIDS	3 (4.1)	4 (5.1)	7 (4.6)	-

ETB: Ethiopian Birr; SD: standard deviation

**Clinical characteristics and diagnostic investigation:** CT-scan was done for 127 (83.0%) patients while the rest of the patients were assessed clinically for stroke based on WHO criteria. Based on CT-scan results, 61 (48.0%) patients had ischemic stroke and 66 (52.0%) had a hemorrhagic stroke. Considering those diagnosed clinically and with brain CT-scan, the overall proportion of ischemic and hemorrhagic stroke was 74 (48.4%) and 79 (51.6%) respectively. Common locations of hemorrhagic stroke were basal ganglia by 35 (53.0%), followed by pones/brainstem 12 (18.2%) and thalamus by 10 (15.2%). Only 14 (9.2%) of stroke patients arrived the hospital within 4.5 hours of stroke symptom onset. The majority 74 (65.3%) of the patients presented after 12 hours of stroke symptom onset. Ninety-three (60.8%) of the patients had a GCS of 13-15 on admission and two-thirds of patients (66.7%) had severe and very severe stroke conditions based on NIHSS score. Almost all patients 144 (94.1%) had hemiparesis or hemiplegia on admission and 71 (46.4%) had headaches and aphasia on admission (Table 2).

**Table 2:** clinical presentations of adult stroke patients admitted to JMC, Ethiopia

Variables	Ischemic Stroke (n=74)	Hemorrhagic stroke (n=79)	All patients (n=153)	p-value
Symptom onset to hospital arrival*	18.5 (10.5-29.5)	15.0 (7.0-24.0)	16 (8.5-27.5)	0.125
≤4.5 hours	7 (9.5)	7 (8.9)	14 (9.2)	0.366
4.51-12 hours	15 (20.3)	24 (30.4)	39 (25.5)	
12.01-24 hours	26 (35.1)	29 (36.7)	55 (35.9)	
24.01-48 hours	26 (35.1)	19 (24.1)	19 (29.4)	
**GCS on admission**				
≤8	6 (8.1)	11 (13.9)	17 (11.1)	0.131
9-12	26 (35.1)	17 (21.5)	43 (28.1)	
13-15	42 (56.8)	51 (64.6)	93 (60.8)	
**NIHSS at hospital arrival**				
≥13	22 (29.7)	29 (36.7)	51 (33.33)	0.360
<13	52 (67.5)	50 (63.3)	102 (66.67)	
Systolic blood pressure	148.6±30.2	163.2±30.4	156.2±31.1	0.004*
Diastolic blood pressure	89.5±20.8	101.1±23.1	95.5±22.7	0.001*
Heart rate	90.6±19.3	86.1±19.8	88.3±19.6	0.161
**Clinical presentations**				
Headache	31 (41.9)	40 (50.6)	71 (46.4)	0.279
Aphasia/dysphasia	43 (58.1)	28 (35.4)	71 (46.4)	0.005*
Hemiparesis/hemiplegia	67 (90.5)	77 (97.5)	144 (94.1)	0.069
Slurred speech/dysarthria	15 (20.3)	13 (16.5)	28 (18.3)	0.542
Facial palsy	30 (40.5)	23 (29.1)	53 (34.6)	0.138
Vomiting	12 (16.2)	25 (31.6)	37 (24.2)	0.026*
Incontinence	21 (28.4)	14 (17.7)	35 (22.9)	0.117
Change in mentation	30 (40.5)	34 (43.0)	64 (41.8)	0.754
Others†	19 (25.7)	11 (13.9)	30 (19.6)	0.067
Brain CT-Scan	61 (82.4)	66 (83.5)	127 (83.0)	0.403

* Expressed as the median and interquartile range (IQR); †Dysphagia, Vertigo/dizziness; CT: Computed tomography; GCS: Glasgow coma scale; NIHSS: National Institute of Health Stroke Score

**Stroke management:** antihypertensive medications were given to about 80 (52.3%) of the patients and these medications were most commonly prescribed for patients who presented with hemorrhagic stroke compared to those presenting with ischemic stroke (p-value <0.001). Statins and aspirin were prescribed for 72 (47.1%) and 68 (44.4%) of the patients respectively. A small number of hemorrhagic patients received aspirin and heparin because of clinical diagnosis and later discontinued after CT-scan result. At discharge, antihypertension medications were prescribed for 81 (53.6%) followed by statins 60 (39.7%) and aspirin 59 (39.1%) (Table 3).

**Table 3:** in-hospital and discharge medication by stroke subtypes

Medications, n (%)	Ischemic stroke (n=74)	Hemorrhagic stroke (n=79)	All patients (n=153)	p-value
**In-hospital medications**				
Antihypertensive*	24 (32.4)	56 (70.9)	80 (52.3)	<0.001
Statins	65 (87.8)	7 (8.9)	72 (47.1)	<0.001
Aspirin	62 (83.8)	6 (7.6)	68 (44.4)	<0.001
Warfarin	5 (6.8)	-	5 (3.3)	-
Any heparin	13 (17.6)	5 (6.3)	18 (11.8)	0.031
Antibiotics	4 (5.4)	11 (13.9)	15 (9.8)	0.077
**Discharge medications**				
Antihypertensive*	27 (37.5)	54 (68.4)	81 (53.6)	<0.001
Statin	55 (76.4)	5 (6.3)	60 (39.7)	<0.001
Aspirin	59 (76.4)	-	59 (39.1)	-
Warfarin	4 (5.6)	-	(2.6)	-

* Thiazide diuretics, angiotensin converting enzyme inhibitors, calcium channel blockers

**In-hospital and 28-day outcome:** the most common in-hospital complications identified were bladder incontinence 53 (34.9%), aspiration pneumonia 32 (21.1%), and increased intracranial pressure (ICP) 30 (19.7%). Except for increased ICP which was more common in hemorrhagic stroke patients (p-value= 0.027), there was no statistically significant difference regarding complications between stroke subtypes. The median length of hospital stay was 7.6 days. The overall in-hospital mortality rate was 26 (17%) and the all-cause 28-day mortality rate was 39 (25.5%). There was no significant difference between stroke subtypes (Table 4). At 28-day follow-up 39 (25.5%) and 43 (28.1%) had moderate disability (mRs=3) and severe disability (mRs=4-5) respectively.

**Table 4:** in-hospital and 28-day treatment outcomes of stroke patients admitted to JMC, Ethiopia

Clinical outcomes, n (%)	Ischemic stroke (n=74)	Hemorrhagic stroke ( n=79)	All patients N (153)	p-value
Urinary/bladder incontinence	27 (37.0)	26 (32.9)	53 (34.9)	0.598
Aspiration pneumonia	13 (17.8)	19 (24.1)	32 (21.1)	0.346
Seizure	10 (13.7)	9 (11.4)	19 (12.5)	0.668
Increased ICP	9 (12.3)	21 (26.6)	30 (19.7)	0.027*
Dysphagia	8 (11.0)	5 (6.3)	13 (8.6)	0.308
In-hospital death	11(14.9)	15(19.0)	26 (17.0)	0.497
Length of hospital stay‡	7.7 (3-10)	7.4 (3-10)	7.6 (3-10)	0.924
28-day death	20 (27.0)	19 (24.1)	39 (25.5)	0.673
MRS at 28-day				
0-2	12 (16.2)	20 (23.3)	32 (20.9)	0.295
3	23 (31.1)	16 (20.3)	39 (25.5)	
4-5	19 (5.7)	24 (30.4)	43 (28.1)	
6	20 (27.0)	19 (24.1)	39 (25.5)	

ICP: intracranial pressure; mRs: modified Rankin scale; ‡Expressed as the median and interquartile range (IQR); *statistically significant at p-value <0.05

**Factors associated with 28-day stroke mortality:** on univariate analysis, eight variables (sex, age, residence, Glasgow coma Scale (NIH), heart rate, aspiration pneumonia, and increased intracranial pressure) had p-value < 0.25. Among these variables, only three variables showed statistically significant association (p-value < 0.05) on multivariable cox-regression. Rural residence, development of aspiration pneumonia, and increased ICP were associated with 28-day mortality. The rate of 28-day mortality in patients from rural areas was 3.5 times higher than in patients who were from urban areas (aHR= 2.93, 95% CI=1.46-5.81). Patients who develop aspiration pneumonia had 6.6 times more likely to have 28-day mortality (aHR= 6.57, 95% CI=3.16-13.66). Similarly, stroke patients complicated with increased ICP had about a three-fold increase in the 28-day mortality rate compared to those patients who did not develop increased ICP (aHR= 3.27, 95% CI=1.56-6.86) (Table 5).

**Table 5:** factors associated with 28-day mortality among stroke patients admitted to JMC, Ethiopia

Variables		28-day status		Univariate analysis		Multivariate analysis	
		Dead (n=39)	Alive (n=114)	HR (95% CI)	p-value	HR (95% CI)	p-value
Age ‡		60.6±17.6	55.9±13.8	1.02 (0.99-1.04)	0.124	1.02 (1.00-1.04)	0.046*
Sex	Male	16 (41.0)	65 (57.0)	058 (.31-1.10)	0.095	0. 98 (0.48-2.02)	0.960
	Female	23 (59.0)	49 (43.0)	-	-	-	-
Residence	Urban	13 (33.3)	79 (69.3)	3.5 (1.80-6.84)	<0.001	2.93 (1.46-5.81)	0.002*
	Rural	26 (66.7)	35 (30.7)	-	-	-	-
GCS	<8	10 (25.6)	7 (6.1)	5.53 (2.48-12.34)	<0.001	1.57(0.58-4.26)	0.374
	9-12	14 (35.9)	29 (25.4)	2.24 (1.08-4.65)	0.03	1.22 (0. 55-2.68)	0.622
	13-15	15 (38.5)	78 (68.4)	-	-	-	-
NIH	<13	18 (46.2)	84 (73.7)	2.78 (1.48-5.22)	0.001	0.85 (0.321-2.22)	0.732
	≥13	21 (53.8)	30 (26.3)	-	-	-	-
Heart rate ‡		96.9±21.4	85.4±18.2	1.03 (1.01-1.04)	0.001	1.004 (0.987-1.02)	0.639
Aspiration pneumonia	Yes	24 (61.5)	8 (7.0)	10.22 (5.31-19.68)	<0.001	6.57(3.16-13.66)	<0.001
	No	15 (38.5)	106 (93.0)	-	-	-	-
Increased ICP	Yes	19 (48.7)	11 (9.6)	6.03 (3.20-11.36)	<0.001	3.27 (1.56-6.86)	0.002*
	No	20 (51.3)	103 (90.4)	-	-	-	-

CI: confidence interval; GCS: Glasgow Coma Scale; HR: hazard ratio; ICP: Increased intracranial pressure; NIHSS: National Institute of Health Stroke Score; Expressed as a mean and standard deviation; *statistically significant at p-value <0.05.

## Discussion

The present study aimed at examining clinical characteristics, in-hospital complications, 28-day mortality and physical disability among stroke patients. More than half of the patients presented with the hemorrhagic stroke while nearly two third of the cases had hypertension as the most common comorbidity. Two-thirds of the patients presented after 12 hours of stroke onset. The use of evidence-based medications was suboptimal and none of the ischemic stroke patients receives reperfusion therapy primarily due to the unavailability of thrombolytic therapy. Stroke admission at the hospital was associated with a very high all-cause mortality rate at 28 days and significant disability (mRs= 4-5).

In our study, hemorrhagic stroke accounted for more than half (51.6%) of total stroke patients. This finding is supported by prior literature from sub-Saharan Africa which reported that the prevalence of hemorrhagic stroke is almost equal to or greater than that of ischemic stroke [8,11,13,22]. However, the proportion of ischemic stroke recorded in the present study was generally higher than that reported by studies from different regions of the world [16,17,23]. Although the exact reason for this discrepancy is not known, the fact that hypertension was a common risk factor in our study participants might contribute to the high prevalence of hemorrhagic stroke in the present study. Like most other studies from Africa mentioned above, our study was limited to hospitalized patients. As a result, the proportion of haemorrhagic stroke may not represent the real prevalence of the stroke subtype due to the fact that patients with ischemic stroke are likely to have milder clinical symptoms and may as a result not even visit healthcare facilities. Although most stroke complications are preventable, a high number of stroke patients in this study developed neurologic and medical complications. Common complications that occurred during this study were urinary/bladder incontinence, aspiration pneumonia, increased ICP, and seizure. These complications were also reported by previous studies as common neurological and medical complications of stroke [14,16,24]. Prevention and early management of stroke complications are an area that needs the attention of clinicians as it is associated with poor prognosis, prolonged hospital stay, and high healthcare cost.

Regarding the management of stroke, antihypertensive, statins, and antiplatelets were the most commonly used medications both at admission and discharge. Antihypertensive medications were commonly used among hemorrhagic stroke patients while statins and antiplatelets were commonly used in ischemic stroke patients. However, a few hemorrhagic stroke patients also received antiplatelet and anticoagulants before confirmation of stroke subtype by brain CT scan. This finding was consistent with previous studies from Ethiopia [24]. None of the ischemic stroke patients received thrombolytic therapy due to the unavailability of these medications. Even if these medications were available, most of the patients were not illegible for thrombolytic medications due to prolonged pre-hospital delay. Overall, the management of stroke in the setting is suboptimal due to a lack of standard treatment protocol, unavailability of thrombolytic medications, prolonged prehospital delay, and lack of access to CT scanner, proper neurological evaluation, and nursing care.

The in-hospital mortality rate and 28-day mortality rate were (17.0%) and (25.5%) respectively in the present study. These findings were almost comparable to a previous study conducted in Kenya [23] that reported a 28-day mortality rate of 26.7%, and a study done in Ethiopia [25] and Nigeria [26] that reported 30-day mortality rate of 29.3% and 28.7% respectively. However, the mortality rate recorded in this study is significantly higher compared to reports from high-income countries [27,28] which reported a 30-day mortality rate of about 14%. This significant outcome disparity might be due to a lack of evidence-based medications, limited experienced neurologists and neurosurgeons, lack of stroke rehabilitation centres, limited access to diagnostic modalities, and prolonged pre-hospital delay.

In this study, rural residence, increased ICP, and aspiration pneumonia was significantly associated with a 28-day mortality rate. Similarly, Beyene N *et al*. [14] and Walelgn N *et al*. [18] reported that older age and development of aspiration pneumonia significantly associated with poor treatment outcomes. In addition, a study conducted by Fekadu *et al*. [25] also showed that increased ICP/brain oedema was an independent predictor of 30-day mortality. In our study, patients from rural areas had about a three-fold increase in 28-day mortality. The difference in mortality among the rural versus the urban residents might be because patients from the rural areas had prolonged pre-hospital delay, and low socioeconomic status and the majority of them had no formal education. These factors were reported by Kafale B *et al*. [17] as predictors of poor outcomes among stroke patients.

This study is one of the few prospective studies on stroke in Ethiopia that provides preliminary data on mortality and disability that can inform stroke management strategies and interventions required to decrease morbidity and mortality associated with stroke. However, the present study has also limitations such as sample size, a short duration (28 days) of follow up and not including important outcomes such as quality of life, cost, and standard of care. Therefore, a prospective registry of a large number of stroke patients is important to evaluate the relevant clinical and economic outcomes of stroke in Ethiopia and other SSA.

## Conclusion

More than half of the patients presented with hemorrhagic stroke and hypertension was the most common comorbid condition identified in about two-thirds of the study participants. The majority of stroke patients presented to the hospital after 12 hours of symptom onset. Overall, in-hospital management of stroke was suboptimal and none of the ischemic stroke patients received thrombolysis with recombinant tissue plasminogen activator (rtPA). The all-cause 28-day mortality and physical disability were significantly high.

### What is known about this topic

- *sub-Saharan African countries are facing a sharp increase in stroke incidence and mortality;*
- *Stroke affect the young and productive age group in Ethiopia and other sub-Saharan Africa.*

### What this study adds

- *Hypertension was the most common risk factor identified among stroke patients*;
- *Most of the patients presented after 12 hours of stroke symptoms onset*;
- *28-day mortality and physical disability rate of stroke were high in the study setting*.
